# Prenatal Diagnosis of Chromosome Abnormalities: A 13-Year Institution Experience

**DOI:** 10.3390/diagnostics2040057

**Published:** 2012-11-19

**Authors:** Carmen Comas, Mónica Echevarria, María Ángeles Rodríguez, Ignacio Rodríguez, Bernat Serra, Vincenzo Cirigliano

**Affiliations:** 1 Department of Obstetrics and Gynecology, Institut Dexeus, Fetal Medicine Unit., Gran Via Carles III, 71-75, 08028 Barcelona, Spain; E-Mails: monech@dexeus.com (M.E.); marrod@dexeus.com (M.A.R.); nacrod@dexeus.com (I.R.); silnun@dexeus.com (B.S.); 2 Molecular Genetics, LABCO Diagnostics, Gran Via Carles III, 71-75, 08028 Barcelona, Spain; E-Mail: vc@general-lab.com

**Keywords:** screening, prenatal invasive test, chromosome abnormality, down syndrome

## Abstract

*Objective*: To analyze trends in screening and invasive prenatal diagnosis of chromosome abnormalities (CA) over a 13-year period and correlate them to changes in the national prenatal screening policy. *Methods*: We retrospectively reviewed Down syndrome (DS) screening tests and fetal karyotypes obtained by prenatal invasive testing (IT) in our fetal medicine unit between January 1999 and December 2011. *Results*: A total of 24,226 prenatal screening tests for DS and 11,045 invasive procedures have been analyzed. Over a 13-year period, utilization of non-invasive screening methods has significantly increased from 57% to 89%. The percentage of invasive procedures has declined from 49% to 12%, although the percentage of IT performed for maternal anxiety has increased from 22% to 55%. The percentage of detected CA increased from 2.5% to 5.9%. Overall, 31 invasive procedures are needed to diagnose 1 abnormal case, being 23 procedures in medical indications and 241 procedures in non-medical indications. *Conclusions*: Our experience on screening and invasive prenatal diagnostic practice shows a decrease of the number of IT, with a parallel decline in medical indications. There is an increasing efficiency of prenatal screening program to detect CA. Despite the increasing screening policies, our population shows a growing request for prenatal IT. The a priori low risk population shows a not negligible residual risk for relevant CA. This observation challenges the current prenatal screening strategy focused on DS; showing that the residual risk is higher than the current cut-off used to indicate an invasive technique.

## 1. Introduction

Current strategies for prenatal diagnosis of chromosome abnormalities (CA) focus on the selection of high-risk pregnancies, to perform invasive diagnostic procedures. Advanced maternal age was the main risk factor for Down syndrome (DS) and prenatal invasive procedure was routinely performed in women over 35 years [1,2]. However, this strategy only detects about half of DS cases, with many women undergoing an invasive procedure for each CA detected.

Second trimester prenatal screening, including maternal age, maternal serum levels of alpha-fetoprotein (AFP) and human chorionic gonadotropin (hCG), was introduced in our country at the end of the 80s. Biochemical parameters together with maternal age increased the detection rate for DS to 60% for a 5% false-positive rate (FPR) [3]. During the last decade, first trimester prenatal screening has been developed; this includes maternal age, maternal serum concentration of free β-hCG and pregnancy-associated plasma protein (PAPP-A), combined with one ultrasound measurement, the nuchal translucency (NT). Implementation of early combined screening should enable to identify up to 90% of DS cases for a 5% FPR [4,5]. Most protocols recommend invasive testing when the estimated risk for DS is higher than 1/270. Currently, DS screening is the most common and costly prenatal detection policy.

The aim of this descriptive study was to analyze trends in screening and invasive prenatal diagnosis over a 13-year period in relation to changes in the national prenatal screening policy. 

## 2. Objectives

To evaluate the impact of the introduction and expansion of the early aneuploidy screening program on the genetic invasive procedures in our institution. To describe the efficiency of the invasive prenatal diagnostic practice in a single-center over a 13-years period, according to indication for referral.

## 3. Methods

This is a retrospective cohort study of all consecutive pregnancies undergoing prenatal screening for DS and genetic invasive procedures (amniocentesis or chorionic villus sampling—CVS) between January 1999 and December 2011 at Dexeus Institut, a private center with a fetal medicine national referral unit.

According to Spanish health policies, second trimester screening for DS was carried out during the first 4 years and first trimester combined screening from 2003 onwards, either in a one-step (blood tests and NT measurements adjusted to crown to rump length—CRL 45–85 mm) or two-step strategy (blood test adjusted to CRL 15–84mm, NT measurement adjusted to CRL 45–85 mm). NT measurements were performed by experienced sonographers. The maternal serum biochemistry was measured using the Kryptor analyzer (Brahms Diagnostica). For combined risk calculation the SBP-software was used, a commercial accredited national software widely used in our geographic area. Cytogenetic study was recommended when combined risk index was higher than 1/270 at screening time. Our healthcare guidelines have traditionally recommended screening for DS below 35, but this cut-off has been progressively moved to 38 in 2004 and from January 2009 onwards to all population.

Referral indications for invasive procedures were divided in medical and non-medical (anxiety). Medical indications were advanced maternal age (AMA)(≥35 years before 2004, ≥38 years from 2004 to January 2009), combined first-trimester or biochemical second-trimester screening for DS > 1/270, abnormal fetal ultrasound (including increased NT—over 99 centile—before the introduction of the first-trimester screening, major fetal abnormality—fetal structural abnormality or increased nuchal fold thickness- and soft ultrasound markers), family history (balanced rearrangements, previous aneuploidy, monogenic diseases), other medical indications (pregnancy obtained by *in vitro* fertilization and intracytoplasmic sperm injection—IVF-ICSI, isoimmunization, suspected fetal infection, CA confirmations, pre-implantation genetic diagnosis—PGD confirmations, *etc.*). Cases with more than one referral reason were classified according to the main clinical indication, following the order of priority: parental chromosome rearrangement, ultrasound abnormalities, increased NT, high-risk prenatal screening, previous aneuploidy and soft ultrasound markers. Invasive procedures for parental distress in women under the AMA criteria (<35 years before 2004, <38 years from 2004 to January 2009 and at any maternal age without medical criteria from January 2009 onwards) were classified as non-medical indication (anxiety). Follow-up was performed until delivery or termination of pregnancy.

Depending on their clinical relevance, CA were classified into irrelevant (balanced familiar translocations, inversions and familial markers), of unknown relevance (balanced translocations and markers of unknown origin) and clinically relevant (autosomal trisomies and monosomies, sex CA, deletions and duplications, unbalanced translocations, triploidies, tetraploidies and de novo markers). In the latter group, several sex CA of controversial prenatal relevance were individually analyzed. We discriminate less severe (47, XXX and 47, XYY karyotypes) from more severe sex CA (Turner and Klinefelter syndromes). 

We define the efficiency of genetic invasive testing as the index “number of IT needed to detect 1 relevant CA”, overall and adjusted for referral indication and maternal age. Residual risk of relevant CA was defined as the number of IT per diagnosis in a priori low risk population. Frequencies between groups were compared using the Chi-square test for trend and Fisher test. P-values of <0.05 were considered significant. All analyses were conducted with the statistical package SPSS 15.0.

## 4. Results

### 4.1. Impact of Increased Utilization of Aneuploidy Screening on Genetic Invasive Tests

A total of 24,226 prenatal screening tests for DS (18,785 combined first trimester tests) and 11,045 invasive procedures (10,426 amniocentesis and 619 CVS) have been performed in the study period. A total of 10,540 procedures were performed in singleton, 490 in twin pregnancies (482 amniocentesis and 8 CVS) and 15 in triplets (amniocentesis). Conclusive cytogenetic results were obtained in 10,993 cases (99.5%). Overall, DR of DS by early screening program was 89.9% for a FPR of 6.2%.

**Figure 1:**
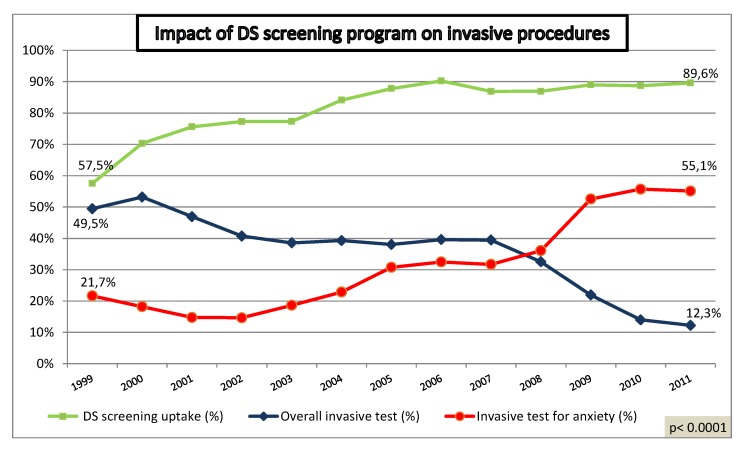
Impact of Down syndrome (DS) screening program on invasive procedures.

**Figure 2:**
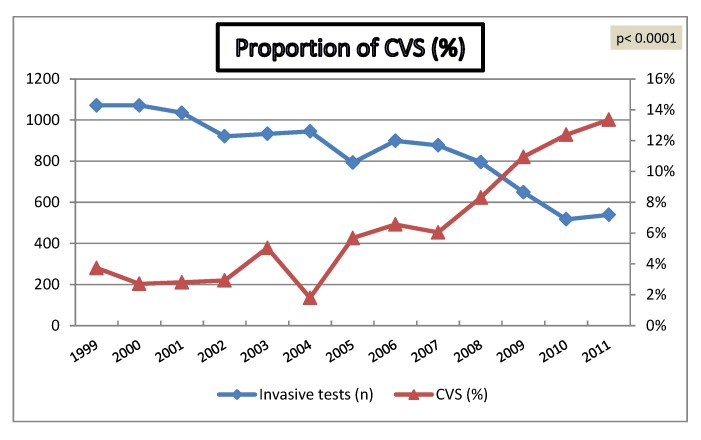
Annual proportion of chorionic villus sampling (CVS).

Over a 13-year period, utilization of non-invasive screening methods has significantly increased from 57% to 89% (p < 0.0001). The percentage of invasive procedures has declined from 49% to 12% (p < 0.0001), although the percentage of tests performed for maternal anxiety has increased from 22% to 55% (p < 0.0001) (Figure 1). The proportion of CVS has progressively increased from 3% to 13% (p < 0.0001) (Figure 2). 

### 4.2. Chromosomal Abnormalities

**Figure 3:**
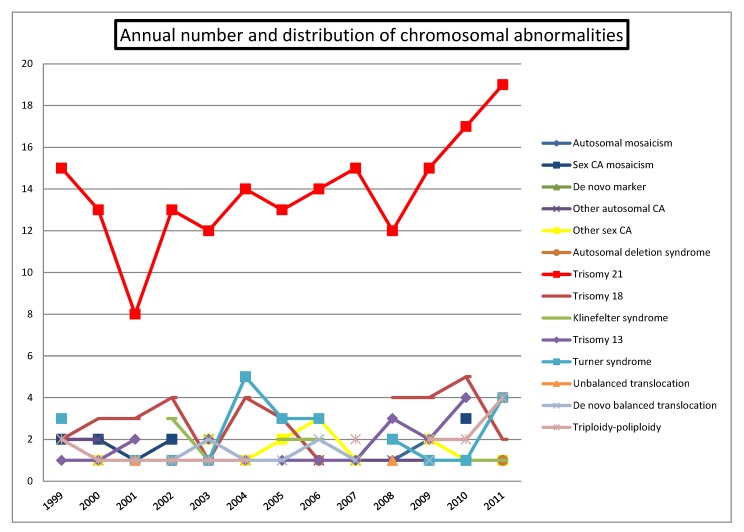
Annual number and distribution of chromosome abnormalities.

**Figure 4:**
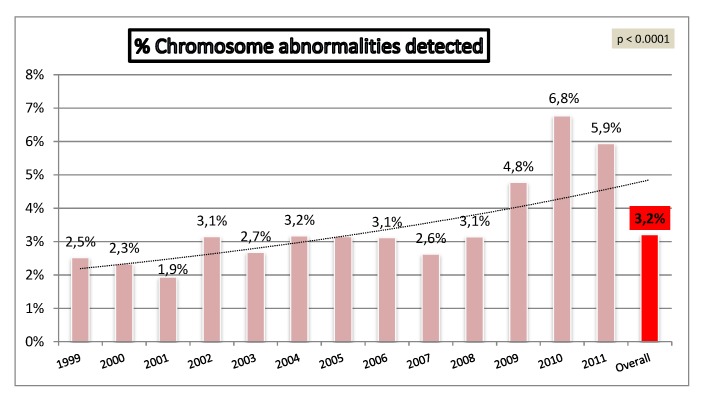
Annual detection of chromosome abnormalities.

A total of 414 CA were detected by conventional cytogenetic analysis (3.8%), 355 of which were classified as clinically significant (Table 1). Figure 3 shows the distribution of CA across the study with a progressive increase of detected DS cases and a stable figure of other CA over the same period. The percentage of CA detected increased from 2.5% to 5.9% (p < 0.0001) (Figure 4).

**Table 1:** Relevant chromosomal abnormalities according to indication of referral.

Chromosomal abnormality	Medical indication	Non-medical indication (anxiety)	Overall
N overall	7,877	3,129	11,006
Autosomal mosaicism	6	1	7
Sex CA mosaicism	10	1	11
De novo marker	4	1	5
Other autosomal CA	12		12
Other sex CA	11	5	16
Autosomal deletion syndrome	4		4
Trisomy 21	178	2	180
Trisomy 18	36		36
Klinefelter syndrome (47, XXY)	10	2	12
Trisomy 13	19		19
Turner syndrome (45, X)	25		25
Unbalanced translocation	2		2
De novo balanced translocation	8	1	9
Triploidy-Poliploidy	17		17
**Overall**	**342**	**13**	**355**
**Prevalence relevant CA**	**4.34%**	**0.42%**	**3.23%**

n: number of cases. CA: chromosomal abnormality.

### 4.3. Indications for Referral

**Figure 5:**
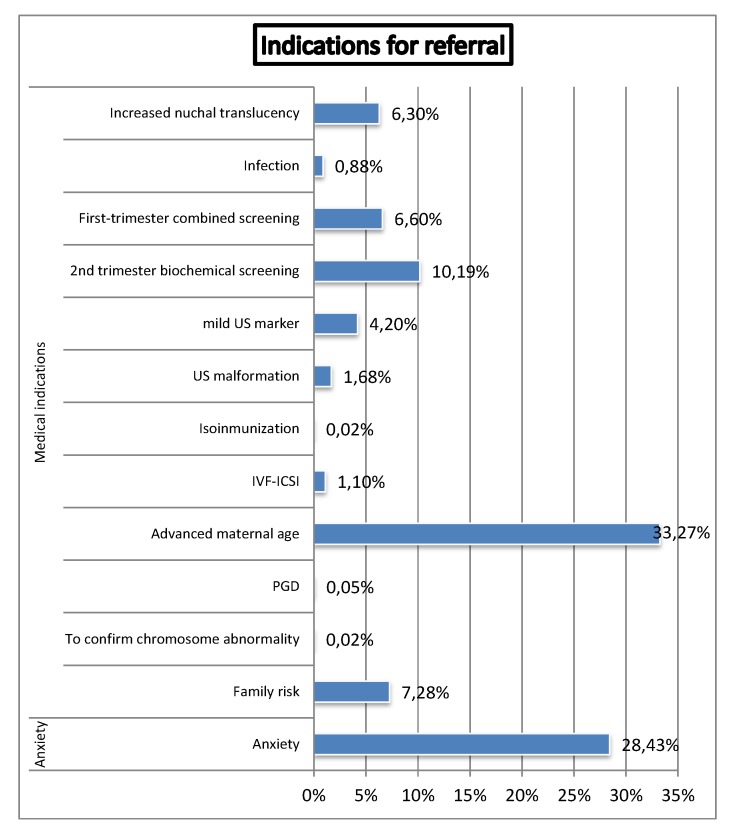
Distribution of indications for referral (IVF-ICSI: *In Vitro* Fertilization-Intra Cytoplasmic Sperm Injection. PGD: Pre-Implantation Genetic Diagnosis).

As shown in Figure 5, main referral indications were: AMA (33.3%), anxiety (28.4%), high-risk at first trimester combined (6.6%) or second-trimester biochemical screening for Down syndrome (10.2%), increased NT (6.3%), major ultrasound abnormality (1.7%), mild ultrasound markers (4.2%), family history (7.3%) and IVF-ICSI (1.1%).

**Figure 6:**
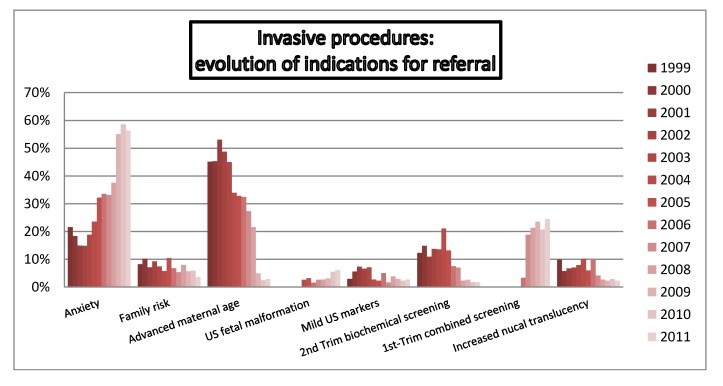
Annual distribution of indications for referral in the overall population.

A shown in Figure 6, distribution of referral indications changed across this period. Initially, AMA was the most common indication for invasive test, been progressively replaced by high risk screening, which become the most common medical indication for invasive procedures. Overall and currently, in our series, non-medical indication was the most frequent reason for invasive test. 

**Figure 7:**
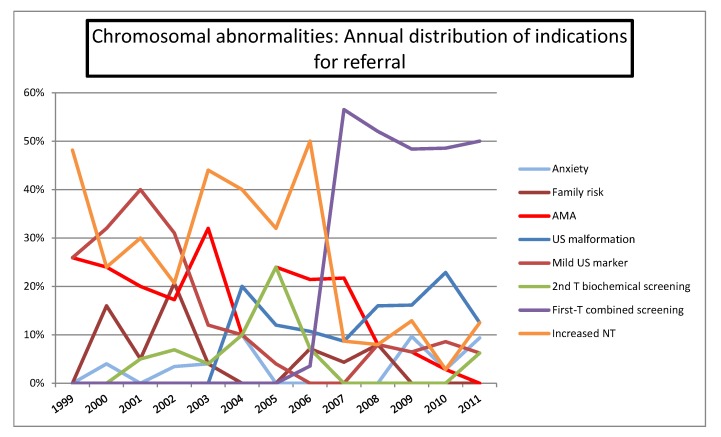
Annual distribution of indications for referral in the chromosomal abnormalities series.

Figure 7 shows the annual distribution of referral indications in the CA group, with an increase of referral for first-trimester combined screening and ultrasound abnormalities, in contrast with the decreasing demand for AMA and increased NT. 

**Figure 8:**
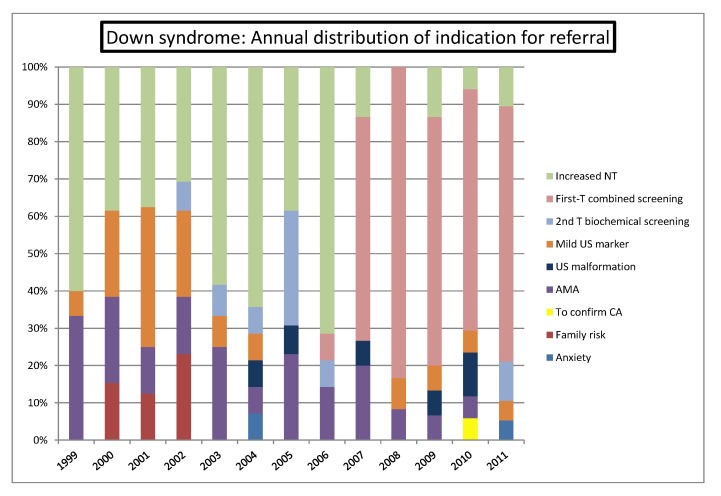
Annual distribution of indications for referral in Down syndrome series.

Focusing in DS cases (n = 180), there was a significant increase of cases detected for first-trimester combined screening indications, in contrast with the decline observed for AMA, increased NT and mild ultrasound markers (Figure 8).

### 4.4. Efficiency of Genetic Invasive Testing According to Indication of Referral

**Figure 9:**
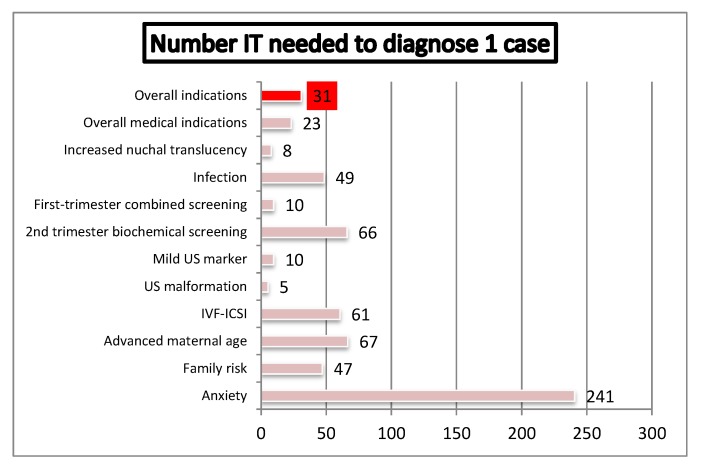
Number of invasive tests needed to diagnose one relevant chromosome abnormality (IVF-ICSI: *In Vitro* Fertilization- Intra Cytoplasmic Sperm Injection).

**Figure 10:**
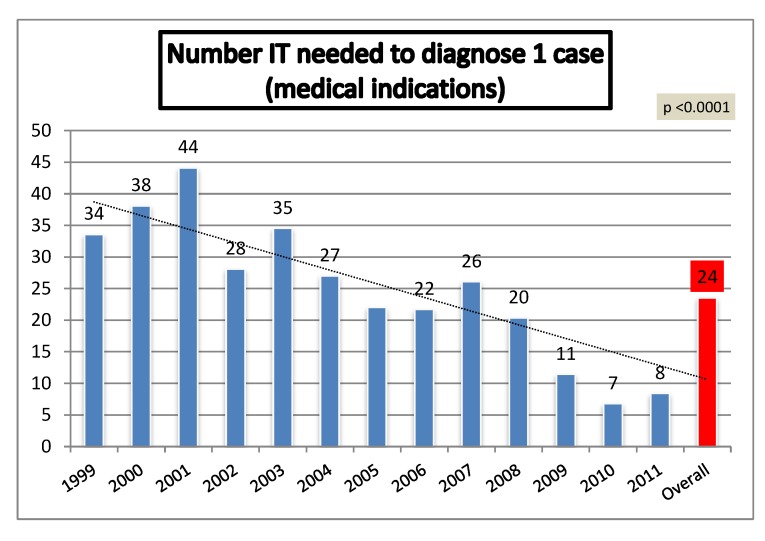
Number of invasive tests needed to diagnose one case of relevant chromosomal abnormality in population referred by medical indication.

The index “IT needed to detect 1 case of relevant CA”, according to referral indication, is showed in Figure 9. Overall, 31 invasive procedures were needed to diagnose 1 case, 23 procedures for medical indications and 241 for maternal anxiety. The lowest number of tests per diagnosis was needed in cases of abnormal ultrasound. In cases referred for medical indications, this index showed a significant decrease across the study (Figure 10, p < 0.0001).

### 4.5. Residual Risk of Chromosomal Anomalies in Low Risk Pregnancies

A total of 3129 procedures (28%) did not have any medical indication (anxiety population). A total of 13 cases (0.42%) of relevant CA were detected, one in a monochorionic twin pregnancy: Two cases of DS were detected, one in a 34 years old women with no ultrasound markers for aneuploidy and one in a 42 years old women with egg donation and a low combined risk for DS; eight cases of sex CA were detected, 5 cases classified as more severe (two cases of 47, XXY, two cases of Turner mosaicism and one case with a Xq22 deletion) and 3 less severe cases (two cases of 47, XXX and one 47, XYY); one *de novo* marker mosaicism (in a monochorionic twin pregnancy), one autosomal mosaicism and one *de novo* balanced translocation were also detected in this group. Overall, 6 cases ended in a termination of pregnancy (TOP) (46%), two Klinefelter syndrome, a de novo marker mosaicism (monochorionic twin pregnancy), one autosomal mosaicism and two DS cases. The remaining pregnancies had a normal follow-up up at delivery.

The index “number of IT needed to detect 1 relevant CA” in this low risk population was 241 (313 if we exclude less severe sex CA). Once adjusted for indication and maternal age, the number of test per diagnosis was 275 under 35 and 210 over this age. This index shows a decreasing trend across the 13-year period (Fisher test, p = 0.05). 

## 5. Discussion

Our series reflects the evolution of prenatal DS screening and genetic invasive diagnostic policy in our institution over a period of 13 years. Several strategies have been introduced along this period to improve the efficiency of the program. The main changes were removing AMA as a single criterion for invasive procedure and introducing the early combined screening for DS. Initially, AMA was the main referral reason for prenatal invasive test, as it was well known that fetal aneuploidies and maternal age are positively correlated [2]. Nowadays, AMA as a single criterion is considered obsolete, as it is included in the prenatal screening for fetal aneuploidies offered to all pregnant women in Spain [6]. Accordingly, our national healthcare guidelines have traditionally recommended invasive testing over 35 and offered DS screening under this age. By the end of 2004, local guidelines have moved the cut-off to 38 years. Recently, since January 2009 onwards, the policy has definitively changed, providing screening to all population. Related to DS screening, second trimester prenatal screening for DS was introduced in our country at the end of the 80s and includes, apart from maternal age, maternal serum levels of AFP and β-hCG. During the last decade, combined first trimester prenatal screening, which includes maternal age, maternal serum concentration of free β-hCG and PAPP-A and NT, has been introduced. 

This series expands our previously published casuistry on aneuploidy screening and genetic invasive test [6]. To our knowledge, this experience is one of the largest Spanish published series of screening and invasive prenatal diagnostic practice. The strength of this study is that we collected data over a long period of time, resulting in a large number of pregnancies from the same center, from which we know the timing of policy changes over the years. A weakness of this study concerns the private character of the center, which make the results not extrapolable to the national health public system and the loss to follow-up after delivery. 

Our prenatal CA screening strategy through a 13-year period currently shows and optimal DS screening uptake, with a clear shift from the second to first-trimester strategy. Moreover, our results show an optimal DS detection, despite the significant decrease in invasive procedures. A similar impact of screening policy for DS has been previously published by other authors in different geographical areas [7,8,9,10].

Our prenatal invasive diagnostic practice shows a significant 50% decrease in number of invasive procedures, with a parallel decline in medical indications. As described in similar experiences [7,8,9,11], the number of samples received tends to decrease substantially across this period, due to implementation of the first trimester screening for DS detection. Also, we highlight a logical shift towards an earlier diagnosis, with an increased CVS practice as a diagnostic test. However, despite the increased CVS proportion though this period, the overall rate of this technique is still low considering the expansion of first-trimester screening to all pregnant women. The conservative attitude of the counselors in our center could be one of the reasons which contributed to this low CVS rate, where some of them still recommend traditional amniocentesis, which is perceived to be “safer”, against CVS in high-risk patients.

Our results also show that medical indications are more efficient in prenatal diagnosis of relevant CA, particularly, ultrasound abnormalities. In relation to the frequency of CA according to the different referral reasons, the most efficient indications (expressed as the number of invasive tests needed to diagnose one case), as expected and described in previous experiences [9,10,11], were ultrasound abnormalities and increased NT, which indicate that ultrasound plays a very important role in the prenatal diagnosis of CA. Moreover, there is an increasing efficiency of prenatal program to detect CA across this period, with a significant reduction of the number of tests needed to diagnose 1 abnormal condition. This observation reflects a better selection of the pregnancies at risk for aneuploidy, with a significant increasing percentage of CA detected. 

In relation to the clinical indications for cytogenetic study, the distribution observed in this study is similar to that obtained in other published series [9,10,11]. Advanced maternal age and positive prenatal screening are the most frequent reasons for referral, although in our series we highlight the high and increasing demand of invasive test without medical criteria (anxiety group). This fact is related to the characteristics of our center, a private center with a fetal medicine national referral unit, where women from the public system often demand for invasive testing despite the low risk at standard screening tests. However, it is important to highlight that the distribution of indications has varied during the period studied. AMA was the main reason during the first period, as it was the only parameter known to be associated with DS. Nevertheless, positive prenatal screening replaced progressively this indication, as it included maternal age and several parameters related to this condition. In relation to indications for referral in the CA series, particularly in DS group, we highlight a significant increase in first-trimester combined screening and ultrasound abnormalities indications, in contrast with the decline indications of AMA, increased NT (as a single marker) and mild ultrasound markers. These logical dynamics are justified by changes in the prenatal screening strategy of aneuploidy during the analysis period.

Previous studies of prenatal cytogenetic series have revealed that the incidence of CA ranges between 1.0% and 9.4% [9,11,12]. We detected relevant CA in 3.2% of cases, which is an incidence similar to that reported in the previous largest review (3.1%) [12]. As expected, the most commonly detected CA were classical autosomal aneuploidies, which represented 64% of the total number of anomalies detected. Among them, DS was the most frequent diagnosed abnormality. This was an expected finding, as trisomy 21 is the autosomal aneuploidy with highest viability and prenatal screening programs are basically focused on its detection. Interestingly, we observe a progressive increase number of diagnosed DS cases across this period, while the frequency of others CA remains relatively stable. We attribute this finding to the current strategy of prenatal screening for aneuploidy, clearly focused on the detection of this condition. In our series, sex CA represent 18%. From them, monosomy X was the most commonly detected abnormality, probably due to the fact that it is the only sex CA associated with ultrasound findings. Interestingly, the percentage of CA detected increases through the period, which reflects a better selection of the pregnancies at risk for this condition.

Despite the expanding screening policies and the improving efficiency of the invasive prenatal strategy, maternal anxiety in low risk pregnant population shows a growing demand for prenatal invasive test. In our population, the percentage of tests performed due to maternal anxiety has significantly increased from 22% to 55%. Interestingly, according to our data, the population a priori at low risk shows a not negligible residual risk for relevant CA. Our data demonstrate a baseline risk of 1/241 for relevant CA. It is true that this index drops to 1/313 if we exclude less severe sex CA. However, at least we dispute the relevance of the prenatal diagnosis of such a type of anomalies. Sex CA, with an incidence of 1 in 400 men and in 650 women, represents one of the most common types of CA observed in human fetuses [13]. Compared with other genetic disorders that result in a shortened lifespan and severe degeneration, the most common symptoms associated with sex CA are neither life-threatening nor severe. Symptoms among the less severe cases (Triple X syndrome and 47, XYY karyotypes) include learning difficulties, particularly verbal skills or language problems, borderline low-to-normal intelligence quotient, and slightly shorter or taller stature. Symptoms among the more severe sex CA (Turner and Klinefelter syndromes) include infertility, cardiac and kidney malformation (in Turner syndrome) and infertility, small testes, and problems with psychological development, lower IQ, and delayed speech development (in Klinefelter syndrome) [14,15,16]. Relative to other autosomal abnormalities, sex CA have been associated to with high abortion rates, despite their nonlife-threatening impact. Several studies pointed to termination of pregnancy rates between 68 and 81% after a prenatal sex CA diagnosis [17,18,19]. In any case, it is clear that the autonomy of the patient and the undoubted advantages of prenatal diagnosis of any abnormality are solid arguments that support the benefit of prenatal diagnosis of this condition.

Moreover, it is presumed that this baseline risk increases as expanding DS screening programs to the whole population. The indication for invasive technique for AMA is currently obsolete, and now, after conventional DS screening, this population is considered at low risk [6]. As we know, maternal age is the only etiological factor whose link with the CA of number (aneuploidy) is now unequivocally recognized [2]. Accordingly, there is some CA other than DS, also related to AMA and with no possibility of prenatal screening. Consequently, this type of CA will have a higher prevalence in this a priori low-risk population.

Furthermore, the residual baseline risk determined in the course of this study only refers to CA detectable by conventional cytogenetic analysis. Array comparative genomic hybridization (aCGH) also allows identification of submicroscopic CA with clinical significance and it has been shown to increase the detection rate of pathogenic CA up to 1–3% regardless of the indication for analysis [20,21,22,23,24,25,26,27]. Focusing on a low-risk population, Lee *et al.* found 0.52% (1/192) baseline risk of submicroscopic genomic imbalance, even in women with uneventful prenatal examination [20]. Routine implementation of these techniques will presumably result in a further increase of the residual risk. Finally, new technologies are appearing in this scenario, challenging our current screening and prenatal strategy to detect fetal aneuploidies. Recently, non-invasive prenatal detection of some aneuploidies by massive or selective parallel sequencing of maternal plasma DNA has been shown to be highly effective, with a detection rate of over 99% and a false positive rate of below 1% [28,29,30]. Indeed, results on larger series are needed; however, the preliminary results with this technology should lead to considering significant changes in the established screening policies of prenatal care.

## 6. Conclusions

Our results show significant changes in the type, number and indications for prenatal invasive procedures. The implementation of the first-trimester combined test in 2004 and the start of a national prenatal screening policy in 2009 have improved the efficacy of prenatal strategy to detect CA. We conclude, however, that despite the expanding screening policies, in our area, the low risk pregnant population shows a growing demand for prenatal invasive testing. This historical analysis enables us to individualize the prenatal counseling related to baseline risk for CA in this low-risk population. Definitively, this observation challenges the current prenatal screening strategy mainly focused on DS, showing that the baseline residual risk for relevant CA is even higher than the current cut-off used to indicate an invasive technique. In this view, prenatal screening policy should ideally not only focus on offering the first-trimester combined test to women of all ages but also on counseling about the baseline residual risk of relevant CA despite the prenatal screening tests result. While the evaluation of larger series is granted, the much higher detection rate of relevant CA with current technologies, even in a priori low-risk groups (>1/100), should open the door to consider significant changes in currently established screening policies in prenatal care. 
